# Predictors of chronic opioid therapy in Medicaid beneficiaries with HIV who initiated antiretroviral therapy

**DOI:** 10.1038/s41598-021-94690-8

**Published:** 2021-07-29

**Authors:** GYeon Oh, Emily S. Brouwer, Erin L. Abner, David W. Fardo, Patricia R. Freeman, Chris Delcher, Daniela C. Moga

**Affiliations:** 1 Department of Epidemiology, University of Kentucky, Lexington, KY USA; 2 Department of Pharmacy Practice and Science, University of Kentucky, Lexington, KY USA; 3 Institute for Pharmaceutical Outcomes and Policy, University of Kentucky College of Pharmacy, Lexington, KY USA; 4 Takeda Pharmaceuticals, Cambridge, MA USA; 5 Sanders-Brown Center on Aging, University of Kentucky, Lexington, KY USA; 6 Department of Biostatistics, University of Kentucky, Lexington, KY USA

**Keywords:** Epidemiology, Risk factors, HIV infections, Pain management

## Abstract

The factors associated with chronic opioid therapy (COT) in patients with HIV is understudied. Using Medicaid data (2002–2009), this retrospective cohort study examines COT in beneficiaries with HIV who initiated standard combination anti-retroviral therapy (cART). We used generalized estimating equations on logistic regression models with backward selection to identify significant predictors of COT initiation. COT was initiated among 1014 out of 9615 beneficiaries with HIV (male: 10.4%; female: 10.7%). Those with older age, any malignancy, Hepatitis C infection, back pain, arthritis, neuropathy pain, substance use disorder, polypharmacy, (use of) benzodiazepines, gabapentinoids, antidepressants, and prior opioid therapies were positively associated with COT. In sex-stratified analyses, multiple predictors were shared between male and female beneficiaries; however, chronic obstructive pulmonary disease, liver disease, any malignancy, and antipsychotic therapy were unique to female beneficiaries. Comorbidities and polypharmacy were important predictors of COT in Medicaid beneficiaries with HIV who initiated cART.

## Introduction

In the US, an estimated 39% to 90% of patients with HIV report chronic pain^1–6^, including general pain, such as head, leg, and back pain; neuropathic pain; and Kaposi sarcoma-related pain^1^. About one-half of these patients are prescribed opioid analgesics^6^. The prevalence of high-dose opioid prescribing is higher in patients with HIV compared to the general population^7,8^*,* and initiation of chronic opioid therapy (COT) was reported to be 1.5 times higher in opioid-naïve people living with HIV compared to those without HIV, after adjusting for demographics and comorbidities^9^. However, several studies have reported that COT confers risks, such as opioid abuse or dependence^10–12^ and poisoning^13^. Especially among patients with HIV, an increased risk of mortality has been reported for those on COT^14^ and high opioid doses^15^.

Although one-quarter of patients with HIV in the US are women^16^, the majority of people included in observational studies examining the association between prescribing opioids and HIV status were men (78.8–98%)^2,7,8,17,18^. However, descriptive studies suggest that women with HIV suffer from more severe pain^6^ and are prescribed more opioids^19^ compared to men with HIV. Moreover, a longitudinal study reported that among patients with HIV, COT was more common among female participants than “never or sporadic [opioid] use”^20^. Thus, inclusion of both men and women is crucial when assessing prescription opioid therapy and related health concerns in patients with HIV.

Therefore, this study aimed to (1) examine the prevalence of COT in Medicaid beneficiaries with HIV; (2) understand the characteristics of patients with HIV who initiate COT; and (3) investigate whether there are sex-specific factors associated with COT among Medicaid beneficiaries with HIV after standard combination anti-retroviral therapy (cART) initiation.

## Methods

### Data source and study population

Medicaid administrative data (2002–2009), obtained from the Centers for Medicaid and Medicare Services, was used for this study. In 2009, 41% of nonelderly adults with HIV in care was covered by Medicaid, which was the largest source of insurance coverage^21^. Beneficiaries may become Medicaid-eligible through several mechanisms, which may depend on the state of residence. Generally, the criteria of Medicaid eligibility are based on low income, pregnancy, and disabilities. This study included the Medicaid Analytic eXtract (MAX) personal summary file (PS), inpatient (IP), prescription (RX), and other services (OT) from four states––Kentucky (KY), Maryland (MD), North Carolina (NC), and Washington (WA). These four states represent different HIV populations during the years of the study––patients with HIV in Maryland are more likely to have acquired HIV through intravenous drug user (IVDU)^22^; Washington has a high proportion of people living with HIV who are men who have sex with men (MSM)^23^; North Carolina has a high proportion of heterosexual patients with HIV^24^; and Kentucky is one of the states with low prevalence and morbidity of HIV^25^.

We included patients with HIV defined as those who filled prescriptions for standard cART on the same date (i.e., cohort entry date) for at least seven days’ supply among Medicaid eligible beneficiaries between 2002 and 2009. Standard cART regimens included two nucleoside reverse transcriptase inhibitors (NRTIs) with either a non-nucleoside reverse transcriptase inhibitor (NNRTI), integrase strand transfer inhibitor (INSTI), or protease inhibitor (PI). The drug name and class were identified from the Medicaid data using national drug codes (NDC) linked with Medispan Generic Product Identifiers (GPI). Inclusion criteria included: (1) age 18–65 years at the cohort entry date; (2) continuous eligibility for at least 180 days prior to the cohort entry date, allowing a one-month gap (i.e., washout period). We excluded beneficiaries who (1) had less than six-months between enrollment and standard cART regimen start date; (2) had COT during the washout period; or (3) had no medical or prescription claim during the washout period. Patient follow-up time was the interval from cART initiation until the earliest of: (1) loss of Medicaid eligibility for > 31 days when multiple enrollments occurred; (2) end of the study period (December 31, 2009). The guideline from CDC defined COT as use of opioids on most days for more than three months^26^; however, there is no standard definition of COT^27^. Several different definitions have been used [e.g., (1) 90 days of continuous use; (2) longer than 90 days use with either 120 + total days of supply or 10 or more prescriptions dispensed in a year; and (3) 90 days of continuous use (accepting ≤ 32 days of the prescription gap), with multiple opioid prescription claims in a six months period] in different studies^2,19,28^. In this study, COT was defined as at least 90 days of continuous opioid analgesic treatment (accepting ≤ 32 days of the prescription gap), with multiple opioid prescription claims in a six months period^28–30^. Opioid analgesics were identified using NDC linked with Medispan GPI (starting with ‘65’).

### Potential predictors

Demographic variables, including age, sex, race, and state of residence, were assessed at the cohort entry date. Age was calculated at cohort entry year based on beneficiaries’ date of birth using PS files. International Classification of Disease, 9th Clinical Modification (ICD-9-CM) codes were used based on existing validated definitions^31–35^ listed anywhere in the IP and OP files to identify beneficiaries’ medical history, and NDCs were used to identify use of medications. Medical history, including diagnoses of cardiovascular disease, hypertension, dementia, chronic obstructive pulmonary disease (COPD), liver disease, diabetes, renal disease, any malignancy (including lymphoma and leukemia, except malignant neoplasm skin), metastatic solid tumor, substance use disorder, Hepatitis C infection, depression, psychotic disorder, back pain, neck pain, arthritis, migraine, opportunistic infection, tobacco use, neuropathy pain, unclassified pain, any fracture, and surgery history, were identified during the washout period. From the RX file, use of medications, including any opioid use, benzodiazepine, gabapentinoid, non-opioid pain medication, including analgesic-anti-inflammatory (GPI starting with ‘66’) and analgesic-nonnarcotic (GPI starting with ‘64’), antidepressant, and antipsychotic were identified during the washout period. Also, polypharmacy, defined as using 5 or more different medications exclusive of the cART regimen, was identified at the cohort entry date. A detailed description of the variables considered in the analysis is included in Table S1.

### Statistical analysis

Baseline characteristics of the beneficiaries included in this analysis were summarized using the mean with standard deviation (SD) for age, median with interquartile range (IQR) for follow-up days, and frequencies with percentages for categorical variables. We used t-tests and Pearson chi-square tests to assess differences between beneficiaries who initiated COT and those who did not. To identify predictors of COT in beneficiaries with HIV who initiated standard cART, we used generalized estimating equations (GEE) with an exchangeable working correlation structure on multivariable logistic regressions to account for within-state correlations among beneficiaries. The final reduced models were fit by backward selection with manually removing the variables with the largest p-value until reaching to the lowest Quasi Information Criterion (QIC). Additionally, multivariable logistic regression models with backward selection (without accounting for state clustering), least absolute shrinkage and selection operator (LASSO) regression using backward selection with the Schwarz Bayesian Information Criterion (SBC)^36,37^, and elastic net logistic regression with cross-validation were used to assess the robustness of the factors identified. Stratified analyses were used to investigate sex-specific predictors of COT. GEE multivariable logistic regression with backward selection was used to identify sex-specific factors associated with COT. Also, multivariable logistic regression models and LASSO regression with SBC using backward selections and elastic net logistic regression model with cross-validation were used to examine the robustness of the sex-specific factors. Adjusted odds ratios (OR_adj_) with 95% confidence intervals (CI) were obtained from the full and reduced models. All data analyses were conducted using SAS 9.4®, and 0.05 was set as the significance level. The study was approved by the University of Kentucky Institutional Review Board (IRB) and the same IRB granted waiver of informed consent due to the nature of the data. All methods were carried out in accordance with relevant guidelines and regulations.

## Results

**Figure 1:**
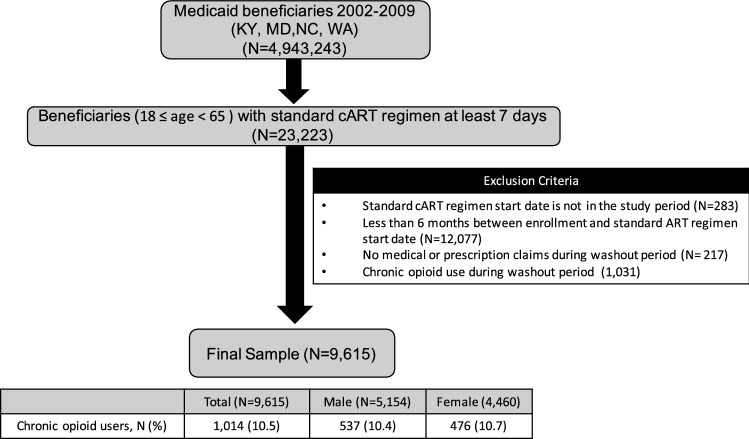
Sample selection flowchart.

In this study, 9,615 Medicaid beneficiaries (mean [SD] age: 41 [10.2] years) with HIV who initiated standard cART were included after applying inclusion and exclusion criteria (Fig. 1). The median (IQR) follow-up years for this sample was 2.15 (0.81–4.48) years and the majority of beneficiaries were male (53.6%) (Table 1). Among the final sample of beneficiaries with HIV (N = 9615), a total of 1,014 beneficiaries (10.5%) initiated COT. The median (IQR) follow-up years was longer in COT initiators (4.06 [2.37–5.84] years) compared to non-initiators (1.90 [0.72–4.24] years). COT initiators were older (43.5 years vs. 40.7 years, *p*-value: < 0.0001), more likely to be white (vs. black) race (26.1% vs. 19.1%, *p*-value: < 0.0001), and had more baseline comorbidities than non-initiators. The most common comorbidities among COT initiators was arthritis and joint pain (final sample: 20.5%; COT: 30.5%; no COT: 19.4%). Polypharmacy was more common among COT initiators than non-initiators (16% vs. 9.4%), and half of COT initiators filled at least one opioid analgesic prescription prior to entering the study (50.9%), whereas approximately 30% of non-initiators filled at least one opioid analgesic prescription (Table 1).

**Table 1:** Characteristics of study sample.

	Total (N = 9615)	Chronic opioid users (N = 1014)	No chronic opioid users (N = 8601)	*p*-value
Age, mean (SD)	41.0 (10.2)	43.5 (7.6)	40.7 (10.4)	< 0.0001
Female	4460 (46.4)	476 (47.0)	3986 (46.3)	0.69
**Race**				
White	1911 (19.9)	265 (26.1)	1646 (19.1)	< 0.0001
Black	7057 (73.4)	686 (67.7)	6371 (74.1)	
Other^a^	266 (2.8)	23 (2.3)	243 (2.8)	
Unknown	381 (4.0)	40 (3.9)	341 (4.0)	
**State**				
Kentucky	407 (4.2)	40 (3.9)	367 (4.3)	0.006
Maryland	4573 (47.6)	508 (50.1)	4065 (47.3)	
North Carolina	3589 (37.3)	334 (32.9)	3255 (37.8)	
Washington	1046 (10.9)	132 (13.0)	914 (10.6)	
**Comorbidities**				
Cardiovascular disease	913 (9.5)	111 (11.0)	802 (9.3)	0.10
Hypertension	1727 (18.0)	236 (23.3)	1491 (17.3)	< 0.0001
Dementia	161 (1.7)	21 (2.1)	140 (1.6)	0.30
COPD	1332 (13.9)	192 (18.9)	1140 (13.3)	< 0.0001
Any liver disease	995 (10.4)	161 (15.9)	834 (9.7)	< 0.0001
Diabetes	695 (10.4)	89 (8.8)	606 (7.1)	0.04
Renal disease	536 (5.6)	65 (6.4)	471 (5.5)	0.22
Any malignancy	377 (3.9)	59 (5.8)	318 (3.7)	0.001
Metastatic solid tumor	59 (0.6)	12 (1.2)	47 (0.6)	0.014
Hepatitis C infection	1069 (11.1)	196 (19.3)	873 (10.2)	< 0.0001
Depression	1750 (18.2)	239 (23.6)	1511 (17.6)	< 0.0001
Psychotic disorder	626 (6.5)	69 (6.8)	557 (6.5)	0.69
Migraine/headache	123 (1.3)	14 (1.4)	109 (1.3)	0.76
Any fracture	209 (2.2)	25 (2.5)	184 (2.1)	0.50
Surgery history	105 (1.1)	13 (1.3)	92 (1.1)	0.54
Opportunistic infection	1863 (19.4)	234 (23.1)	1629 (18.9)	0.002
Alcohol abuse	814 (8.5)	104 (10.3)	710 (8.3)	0.03
Substance use disorder	1850 (19.2)	302 (29.8)	1548 (18.0)	< 0.0001
Tobacco use disorder	834 (8.7)	109 (10.8)	725 (8.4)	0.013
**Pain-related disorder**				
Back pain	790 (8.2)	153 (15.1)	637 (7.4)	< 0.0001
Neck pain	277 (2.9)	45 (4.4)	232 (2.7)	0.002
Arthritis/joint pain	1973 (20.5)	309 (30.5)	1664 (19.4)	< 0.0001
Neuropathy pain	938 (9.8)	163 (16.1)	775 (9.0)	< 0.0001
Unclassified pain	413 (4.3)	62 (6.1)	351 (4.1)	0.003
**Number of medications**				
None	3536 (36.8)	289 (28.5)	3247 (37.8)	< 0.0001
1–4	5113 (53.2)	563 (55.5)	4550 (52.9)	
5 +	966 (10.1)	162 (16.0)	804 (9.4)	
Non-opioid Pain medication	2284 (23.8)	336 (33.1)	1948 (22.7)	< 0.0001
Benzodiazepine	940 (9.8)	191 (18.8)	749 (8.7)	< 0.0001
Gabapentinoid	576 (6.0)	114 (11.2)	462 (5.4)	< 0.0001
Antidepressant	2550 (26.5)	403 (39.7)	2147 (25.0)	< 0.0001
Antipsychotics	1242 (12.9)	180 (17.8)	1062 (12.4)	< 0.0001
Any opioid use	3050 (31.7)	516 (50.9)	2534 (29.5)	< 0.0001

Abbreviation: COPD, Chronic pulmonary disease; COT, Chronic opioid therapy.

All results presented are N (%) unless otherwise noted.

^a^American Indian, Alaskan Native, Hispanic, Asian, Pacific Islander, Native Hawaiian, more than one race.

### Predictors of chronic opioid use

The full model, including all variables are listed in the Table 1, and the reduced model using GEE multivariable logistic regressions are reported in Table 2. Several factors emerged as significant predictors positively associated with COT initiation: age, medical diagnoses (COPD, any malignancy, Hepatitis C infection, opportunistic infection, substance use disorder, back pain, arthritis, neuropathy pain), and use of medications (non-opioid pain medication, benzodiazepine, gabapentinoid, antidepressant, antipsychotic, any opioid, 5 + medication vs. none, 1–4 medications vs. none). Several factors were negatively associated with COT initiation: race (black vs. white; other vs. white) and medical diagnoses (cardiovascular disease, diabetes, psychotic disorder, any fracture, tobacco use disorder, and alcohol abuse) (Table 2).

**Table 2:** Predictors of chronic opioid use vs. no chronic opioid use in Medicaid beneficiaries with HIV.

	Full Model OR_adj_ (95% CI)	Reduced Model OR_adj_ (95% CI)
Age	1.02 (1.01 1.03)	1.02 (1.01, 1.03)
Female	1.06 (0.82, 1.37)	
**Race**		
Black vs. white	0.76 (0.62, 0.93)	0.77 (0.65, 0.91)
Other^a^ vs. white	0.67 (0.58, 0.79)	0.68 (0.59, 0.78)
Unknown vs. white	0.82 (0.67, 1.01)	0.84 (0.67, 1.05)
**Comorbidities**		
Cardiovascular disease	0.78 (0.64, 0.96)	0.77 (0.68, 0.88)
Hypertension	1.11 (0.95, 1.30)	
Dementia	0.89 (0.58, 1.36)	
COPD	1.16 (0.90, 1.50)	1.18 (0.92, 1.51)
Any liver disease	0.93 (0.74, 1.16)	
Diabetes	0.91 (0.78, 1.05)	0.92 (0.82, 1.02)
Renal disease	0.85 (0.58, 1.25)	
Any malignancy	1.26 (1.18, 1.35)	1.28 (1.23, 1.33)
Metastatic solid tumor	1.10 (0.67, 1.83)	
Hepatitis C infection	1.62 (1.41, 1.87)	1.55 (1.47, 1.64)
Depression	0.98 (0.75, 1.30)	
Psychotic disorder	0.71 (0.63, 0.80)	0.70 (0.62, 0.79)
Migraine/headache	0.86 (0.61, 1.21)	
Any fracture	0.70 (0.57, 0.87)	0.70 (0.55, 0.89)
Surgery history	1.04 (0.77, 1.39)	
Opportunistic infection	1.05 (0.98, 1.13)	1.04 (0.98, 1.11)
Alcohol abuse	0.85 (0.69, 1.03)	0.84 (0.66, 1.05)
Substance use disorder	1.62 (1.22, 2.16)	1.62 (1.25, 2.12)
Tobacco use disorder	0.86 (0.74, 1.00)	0.86 (0.78, 1.02)
Back pain	1.44 (1.11, 1.88)	1.46 (1.10, 1.93)
Neck pain	1.02 (0.88, 1.17)	
Arthritis	1.11 (0.98, 1.26)	1.13 (1.01, 1.25)
Neuropathy pain	1.18 (1.05, 1.33)	1.17 (1.05, 1.30)
Unclassified pain	1.04 (0.91, 1.20)	
**Number of medications**		
1–4 medications vs. none	1.35 (1.29, 1.41)	1.34 (1.29, 1.40)
5 + medications vs. none	1.73 (1.44, 2.08)	1.71 (1.40, 2.09)
Non-opioid pain medication	1.15 (0.94, 1.41)	1.16 (0.96, 1.40)
Benzodiazepine	1.62 (1.49, 1.76)	1.62 (1.48, 1.77)
Gabapentinoid	1.33 (1.21, 1.46)	1.33 (1.21, 1.45)
Antidepressant	1.31 (1.06, 1.62)	1.31 (1.10, 1.56)
Antipsychotic	1.09 (0.96, 1.24)	1.09 (0.99, 1.21)
Any opioid use	1.85 (1.59, 2.15)	1.86 (1.61, 2.13)

^a^American Indian, Alaskan Native, Hispanic, Asian, Pacific Islander, Native Hawaiian, more than one race.

*Abbreviation* COPD, Chronic pulmonary disease.

*Note* Generalized estimate equation multivariable logistic regressions, accounting for clustering at the state level, were used and the reduced model was selected by backward selection.

The reduced model without accounting for clustering within states reported similar OR_adj_ with 95% CI, but diabetes, any malignancy, any fracture, opportunistic infection, alcohol abuse, tobacco use disorder, arthritis, COPD, and use of antipsychotic medications were not included in the reduced model without clustering (Table S2). Another reduced model using LASSO reported similar OR_adj_ with 95% CI, but factors, including diabetes, any malignancy, any fracture, opportunistic infection, alcohol abuse, tobacco use disorder, cardiovascular disease, psychotic disorder, and antipsychotic medications were not in the reduced model using LASSO (Table S2). The predictors included in the reduced model using elastic net with cross-validation were more similar to the final model, but having diabetes, opportunistic infection, tobacco use disorder, and antipsychotics were not included (Table S2). After selecting factors by LASSO and elastic net, the factors were entered into the GEE multivariable logistic regression accounting for clustering within states. Thus, considering state as a significant clustering factor in this study, GEE multivariable logistic regression models were selected as our final model.

### Characteristics of the beneficiaries by sex

Female beneficiaries were on average younger than males (mean [SD] age 39.0 [10.4] vs. 42.7 [9.6] years, respectively, *p*-value: < 0.0001), and the median [IQR] follow-up days was slightly longer (*p*-value: < 0.0001) in females (801 [307.5–1624)) than males (759 [287–1646)). The most common comorbidity in both sexes was arthritis and joint pain (male: 20.2%; female: 20.9%). Male beneficiaries had a higher percentage of polypharmacy (11.1%) than females (8.9%) (Table 3).

**Table 3:** Characteristics of study sample by sex.

	Male (N = 5154)			Female (N = 4460)			*p*-value^a^
	Total (N = 5154)	Chronic opioid users (N = 537)	No chronic opioid users (N = 4617)	Total (N = 4460)	Chronic opioid users (N = 476)	No chronic opioid users (N = 3984)	
Age, mean (SD)	42.7 (9.6)	44.3 (7.3)	42.5 (9.9)	39.0 (10.4)	42.5 (7.8)	38.6 (10.6)	< 0.0001
**Race**							< 0.0001
White	1,264 (24.5)	162 (30.2)	1,102 (23.9)	646 (14.5)	102 (21.4)	544 (16.7)	
Black	3517 (68.2)	338 (62.9)	3179 (68.9)	3540 (79.4)	348 (73.1)	3192 (80.1)	
Other^b^	141 (2.7)	15 (2.8)	126 (2.7)	125 (2.8)	8 (1.7)	117 (2.9)	
Unknown	232 (4.5)	22 (4.1)	210 (4.6)	149 (3.3)	18 (3.8)	131 (3.3)	
**State**							< 0.0001
Kentucky	226 (4.4)	28 (5.2)	198 (4.3)	181 (4.1)	12 (2.5)	169 (4.2)	
Maryland	2300 (44.6)	232 (43.2)	2068 (44.8)	2273 (51.0)	276 (58.0)	1,997 (50.1)	
North Carolina	1839 (35.7)	172 (32.0)	1667 (36.1)	1750 (39.2)	162 (34.0)	1588 (39.9)	
Washington	789 (15.3)	105 (19.6)	684 (14.8)	256 (5.7)	26 (5.5)	230 (5.8)	
**Comorbidities**							
Cardiovascular disease	525 (10.2)	61 (11.4)	464 (10.1)	388 (8.7)	50 (10.5)	338 (8.5)	0.01
Hypertension	882 (17.1)	113 (21.0)	769 (16.7)	845 (19.0)	123 (25.8)	722 (18.1)	0.02
Dementia	104 (2.0)	14 (2.6)	90 (2.0)	57 (1.3)	7 (1.5)	50 (1.3)	0.005
COPD	583 (11.3)	76 (14.2)	507 (11.0)	749 (16.8)	116 (24.4)	633 (15.9)	< 0.0001
Any liver disease	580 (11.3)	91 (17.0)	489 (10.6)	415 (9.3)	70 (14.7)	345 (8.7)	0.002
Diabetes	366 (7.1)	38 (7.1)	328 (7.1)	329 (7.4)	51 (10.7)	278 (7.0)	0.60
Renal disease	336 (6.5)	41 (7.6)	298 (6.4)	200 (4.5)	24 (5.0)	176 (4.4)	< 0.0001
Any malignancy	234 (4.5)	31 (5.8)	203 (4.4)	143 (3.2)	28 (5.9)	115 (2.9)	0.001
Metastatic solid tumor	30 (0.6)	8 (1.5)	22 (0.5)	29 (0.7)	4 (0.8)	25 (0.6)	0.67
Hepatitis C infection	633 (12.3)	106 (19.7)	527 (11.4)	436 (9.8)	90 (18.9)	346 (8.7)	< 0.0001
Depression	795 (154)	105 (19.6)	690 (14.9)	955 (21.4)	134 (28.2)	821 (20.6)	< 0.0001
Psychotic disorder	329 (6.4)	31 (5.8)	298 (6.5)	297 (6.7)	38 (8.0)	259 (6.5)	0.58
Migraine/headache	50 (1.0)	6 (1.1)	44 (1.0)	73 (1.6)	8 (1.7)	65 (1.6)	0.004
Any fracture	135 (2.6)	17 (3.2)	118 (2.6)	74 (1.7)	8 (1.7)	66 (1.7)	0.001
Surgery history	43 (0.8)	7 (1.3)	36 (0.8)	62 (1.4)	6 (1.3)	56 (1.4)	0.009
Opportunistic infection	987 (19.2)	116 (21.6)	871 (18.9)	876 (19.6)	118 (24.8)	758 (19.0)	0.54
Alcohol abuse	494 (9.6)	66 (12.3)	428 (9.3)	320 (7.2)	38 (8.10)	282 (7.10)	< 0.0001
Substance use disorder	937 (18.2)	145 (27.0)	792 (17.2)	913 (20.5)	157 (33.0)	756 (19.0)	0.005
Tobacco use disorder	478 (9.3)	61 (11.4)	417 (9.0)	356 (8.0)	48 (10.1)	308 (7.70)	0.02
**Pain-related disorder**							
Back pain	421 (8.2)	88 (16.4)	333 (7.2)	368 (8.3)	64 (13.5)	304 (7.6)	0.88
Neck pain	146 (2.8)	29 (5.4)	117 (2.5)	131 (2.9)	16 (3.4)	115 (2.9)	0.76
Arthritis/ joint pain	1040 (20.2)	164 (30.5)	876 (19.0)	932 (20.9)	144 (30.3)	788 (19.8)	0.38
Neuropathy pain	501 (9.7)	96 (17.9)	405 (8.8)	437 (9.8)	67 (14.1)	370 (9.3)	0.90
Unclassified pain	122 (2.4)	25 (4.7)	97 (2.1)	291 (6.5)	37 (7.8)	254 (6.4)	< 0.0001
**Number of medications**							< 0.0001
None	1811 (35.1)	148 (27.6)	1663 (36.0)	1724 (38.7)	140 (29.4)	1584 (39.8)	
1–4	2773 (53.8)	301 (56.1)	2472 (53.5)	2340 (52.5)	262 (55.0)	2078 (52.2)	
5 +	570 (11.1)	88 (16.4)	482 (10.4)	396 (8.9)	74 (15.6)	322 (8.1)	
Non-opioid pain medication	1087 (21.1)	159 (29.6)	928 (20.1)	1197 (26.8)	177 (37.2)	1020 (25.6)	< 0.0001
Benzodiazepine	530 (10.3)	102 (19.0)	428 (9.3)	409 (9.2)	88 (18.5)	321 (8.1)	0.07
Gabapentinoid	330 (6.4)	58 (10.8)	272 (5.9)	246 (5.5)	56 (11.8)	190 (4.8)	0.07
Antidepressant	1249 (24.2)	188 (35.0)	1061 (23.0)	1301 (29.2)	215 (45.2)	1086 (27.3)	< 0.0001
Antipsychotics	623 (12.1)	76 (14.2)	547 (11.9)	619 (13.9)	104 (21.9)	515 (12.9)	0.01
Any opioid use	1551 (30.1)	261 (48.6)	1290 (27.9)	1498 (33.6)	254 (53.4)	1244 (31.2)	0.0002

All results presented are N (%) unless otherwise noted.

*Abbreviation* COPD, Chronic pulmonary disease.

^a^T-test and Pearson Chi-Square tests were used to assess differences between male and female beneficiaries.

^b^American Indian, Alaskan Native, Hispanic, Asian, Pacific Islander, Native Hawaiian, more than one race.

Among male beneficiaries, the most common comorbidities were arthritis and joint pain for both COT initiators (30.5%) and non-initiators (19%). Antidepressant medication therapy was common for both COT initiators (35%) and non-initiators (23%). Almost half of the male beneficiaries who initiated COT (48.6%) filled a prescription for any opioid during the washout period. Among female beneficiaries, the most common comorbidity was substance use disorder (33.0%) for COT initiators and arthritis and joint pain (19.8%) for non-initiators. Almost half of the female COT initiators filled a prescription for antidepressants (45.2%), whereas 27.3% of female non-initiators filled a prescription for antidepressants. Over half of the female beneficiaries (53.4%) who initiated COT filled a prescription for opioids during the washout period (Table 3). For most pain-related symptoms, no significant differences in the prevalence between males and females were observed (back pain [*p* = 0.88], neck pain [*p* = 0.76], arthritis and joint pain [*p* = 0.38], and neuropathic pain [*p* = 0.90]). However, higher prevalence of unclassified pain (6.5% vs. 2.4%, *p* < 0.0001) and migraine/headache (1.6% vs. 1.0%, *p* = 0.004) were observed in females (Table 3). There was no statistically significant difference (*p*-value: 0.69) in the prevalence of COT between females (10.4%) and males (10.7%). The COT prevalence was significantly higher in female beneficiaries in MD (male vs. female: 10.1% vs. 12.1%, *p*-value: 0.03). However, no statistically significant differences were observed in the other three states (male vs. female, KY: 12.4% vs. 6.6%, *p*-value: 0.052; NC: 9.4% vs. 9.3%, *p*-value: 0.92; and WA: 13.3% vs. 10.2%, *p*-value: 0.19).

### Predictors of COT by sex

**Figure 2:**
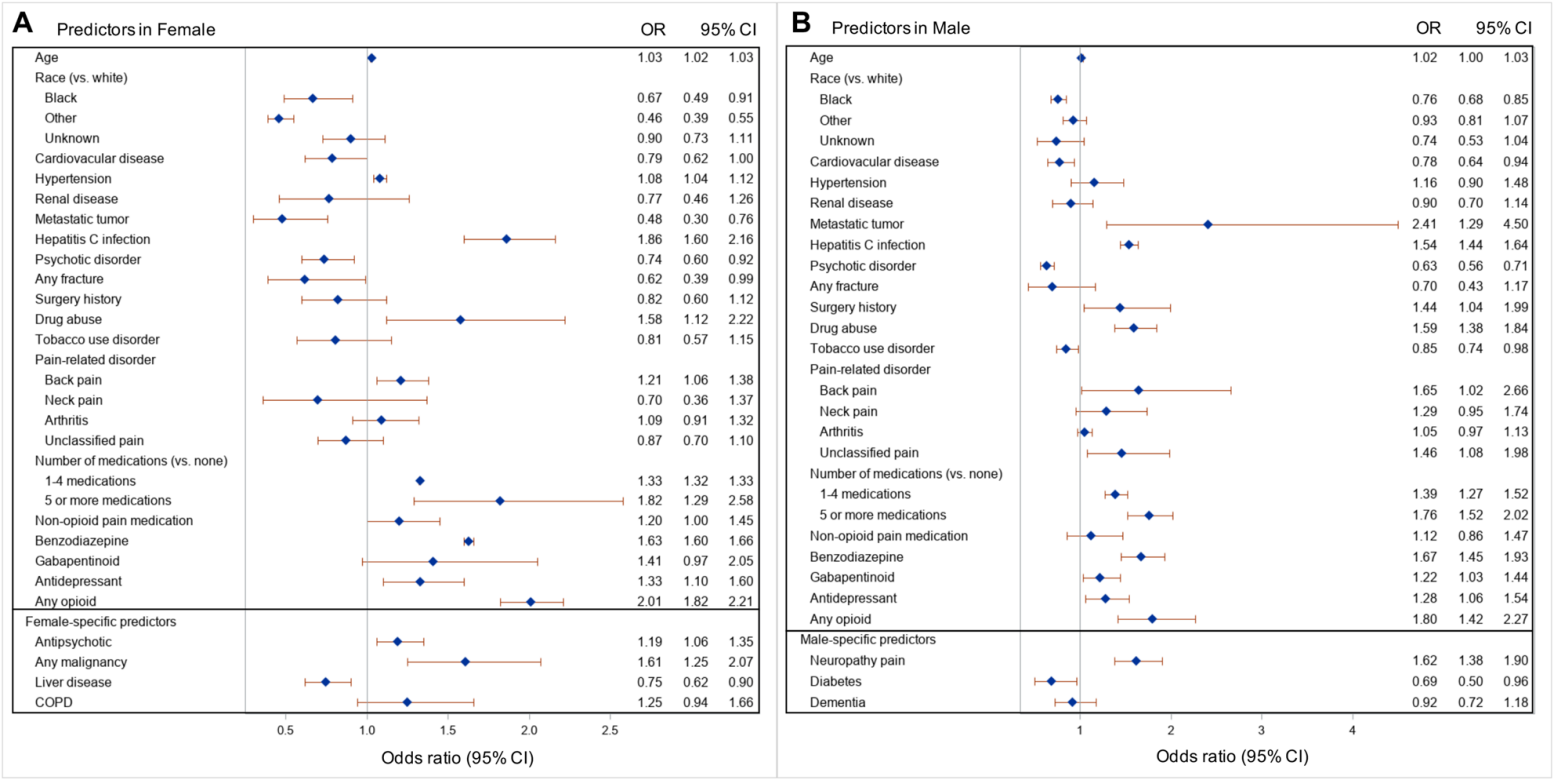
Forest plot presenting the predictors (reduced model) of chronic opioid therapy initiation in female (**A**) and male (**B**) Medicaid beneficiaries with patients with HIV. *Note* Generalized estimating equation multivariable logistic regressions, accounting for clustering at the state level, were used and the reduced model was selected by backward selection.

The predictors of COT (reduced model) in females and males, accounting for within-state correlation, are presented in Fig. 2. The sex-specific full and reduced models with and without accounting for within-state correlation are presented in Tables S3 and S4. Many factors associated with COT were not sex-specific (age; race; having cardiovascular disease, hypertension, renal disease, metastatic solid tumor, Hepatitis C infection, psychotic disorder, any fracture, surgery history, substance use disorder, tobacco use, back pain, neck pain, arthritis, and unclassified pain; polypharmacy; taking non-opioid pain medication, benzodiazepine, gabapentinoid, antidepressant, and any opioid). However, dementia (0.92 [0.72, 1.18]), diabetes (0.69 [0.50, 0.96]), and neuropathic pain (1.62 [1.38, 1.90]) were associated with COT only in males, whereas COPD (1.25 [0.94, 1.66]), liver disease (0.75 [0.62, 0.90]), malignancy (1.61 [1.25, 2.07]), and antipsychotic medication (1.19 [1.06, 1.35]) associated with COT only in females (Fig. 2). Although predictors, including renal disease, surgery history, tobacco use disorder, COPD, neck pain, arthritis, unclassified pain, and use of gabapentinoid in females; and hypertension, renal disease, any fracture, neck pain, arthritis, use of non-opioid pain medication, and dementia in males, were not statistically significant in the final model, they were included due to the improvement of overall fit of the regression model.

## Discussion

This study identified patient-related factors associated with initiation of COT in Medicaid beneficiaries with HIV who initiated cART. The overall prevalence of COT in this study was 10.5% and was higher than a study conducted by Silverberg et al*.* (8.0%) using Kaiser Permanente North California in 2005^19^. However, another cross-sectional study conducted by Merlin et al*.* reported higher prevalence of COT (17%) than our study^2^. The Merlin study included participants with HIV receiving care at the University of Alabama at Birmingham using patient reported outcome data around 2012. These differences could be due to populations with public insurance vs. commercial insurance and data from clinic cohort, and the population of this study was restricted to the beneficiaries with HIV who initiated cART. Also, since there is no standard definition of COT, all of these studies had different definitions for identifying COT. A recent review paper demonstrated that the COT prevalence varied across studies due to different population and COT definition^27^. Also, the study conducted by Merlin et al*.* used more recent data compared to our study.

In our study, the prevalence of COT was similar between males (10.4%) and females (10.7%). This result is inconsistent with previous studies that reported women with HIV suffer from more pain^6^, and that female sex predicts chronic opioid use in Kaiser Permanente members with HIV^19^. This could be due to the different populations included in the study-Medicaid beneficiaries in KY, MD, WA, and NC vs. Kaiser Permanente members in Northern California. However, in our study, the prevalence of COT in WA, which is similar in terms of geography and environment to Northern California, was not significant but higher in males than in females. Among the four states, COT prevalence was higher among female beneficiaries only in MD.

We found that Medicaid beneficiaries with HIV in this study had similar prevalence of pain-related symptoms (back pain, neck pain, arthritis and joint pain, and neuropathic pain) by sex, except higher prevalence of unclassified pain and migraine/headache in females. This result was consistent with a cross-sectional study including residents with HIV in Uganda reported that female sex was positively associated with headache^38^. In populations with similar indications for opioid treatment in males and females, sex differences may be diminished. Therefore, further studies are needed to examine the nature of sex-specific differences in COT among patients with HIV.

The factors we identified as significantly associated with COT initiation were consistent with other studies. A 2005 study that found that factors, including age 40 to 49 years vs. 18 to 39 years, female sex, a Charlson Comorbidity Index score of two or more vs zero, injection drug use history, depression, and substance use disorders, were associated with prevalent COT in Kaiser Permanente members with HIV^19^. A recent study using Veterans Aging Cohort Study survey confirmed that people with HIV with escalating opioid use were more likely to be associated with psychoactive prescribed medications, anxiety symptoms, pain interference, and marijuana use compared to people with HIV with stable, infrequent opioid use^17^. The lower rate of COT initiation among patients with HIV diagnosed with alcohol dependence in this study was also observed from the Merlin study^2^*.* Also, some of the factors associated with COT in the current study also predicted chronic opioid use among older adults enrolled in studies of brain aging and dementia in the National Alzheimer’s Coordinating Center database^39^. Older age; antidepressant, anxiolytic, sedative, or hypnotic agents; and polypharmacy were associated with COT in both studies^39^. This raises a concern that patients with higher medical burden (e.g., more comorbidities, taking benzodiazepines, and polypharmacy) are more likely to be on COT within already vulnerable populations, such as older adults and patients with HIV.

This study showed that polypharmacy was significantly associated with COT in both male and female patients with HIV. However, since polypharmacy is associated with adverse events (e.g., decreased adherence to ARV and increased risk of hospitalization and mortality) in patients with HIV^40–42^, special cautions seem to be needed to prescribe COT in HIV patients with polypharmacy. Especially, we found that treatment with benzodiazepines or gabapentinoids was significantly associated with COT initiation in both sexes. The 2016 guideline provided by the US Centers for Disease Control and Prevention recommend avoiding concurrent use of opioids and benzodiazepines due to the increased risk of fatal overdose^26^, and recently, the US Food and Drug Administration warned about the potential risk of respiratory depression in patients using opioids and gabapentin concurrently^43^. Since our study was conducted well-before these guidelines were released, further studies with more recent data are needed to investigate the trend of concurrent prescriptions of benzodiazepines and gabapentinoids with COT and the potential risks associated with COT in patients with HIV who are concurrently using benzodiazepines or gabapentin.

Most of the comorbidities we assessed were positively associated with COT in patients with HIV, but psychotic disorder was negatively associated in both sexes. In the general population, history of psychotic disorder is reported to be positively associated with chronic opioid use^44,45^. However, this association was not observed in our study of patients with HIV. Because it has been studied that patients with HIV who also have psychotic disorders tend to have higher stimulant use and mortality rate^46,47^, and are positively associated with drug dependence^48^, there is a possibility that providers are careful when prescribing opioids chronically in these patients with HIV. Also, since patients with psychotic disorders have more chance to seek the healthcare provider^49^, it is possible for them to have higher chance of managing pain through non opioid treatment. In contrast, we found that use of antipsychotic medications was positively associated with COT initiation in women. Having concern that use of antipsychotic medications has different effects on COT initiation in patients with HIV by sex, future studies are needed to support our results to conclude that the effect of antipsychotic use on COT initiation in patients with HIV is different between male and female.

Several studies reported that the patients with HIV with lower CD4 + count and advance disease stage had higher prevalence of pain^50–52^. Since we did not have CD4 + counts, we included opportunistic infections which occur more frequently in people with advanced HIV. However, the results in this study indicated that having opportunistic infections was a predictor of COT initiation in patients with HIV but was not significant. This result was consistent to a study conducted by Silverberg et al*.* which reported that the CD4 + , HIV viral load, was not significantly associated with long-term opioid use in 2005^19^.

There are several strengths and limitations in this study. The vast majority of people included in previous studies were male (78.8–98%)^2,7,8,17,18,53^; however, this study was more balanced and included 46.4% of females which allowed us to assess COT in both sexes. We have several unmeasured potential predictors (e.g., clinician characteristics, undocumented opioid use, and use of over-the-counter drugs) due to the limitation of prescription claims data. We used opportunistic infection as a proxy for severity of HIV disease as laboratory data were unavailable. Also, since the recent guidelines of treatment of HIV changed to prescribe cART regardless of CD4 count or viral load, the study sample included in this study will have more severe HIV condition with lower CD4 + cell counts compared to the recent patients with HIV taking cART. Finally, since this study used Medicaid data from four states and restricted to the patients with standard cART, the results may not be generalized to the overall population of patients with HIV.

## Conclusions

In conclusion, one in ten Medicaid beneficiaries with HIV initiated COT from 2002 to 2009. More vulnerable population (e.g., having comorbidities and polypharmacy) were more likely to be associated with COT in patients with HIV. We found that while multiple predictors of COT were shared between males and females, having diabetes, dementia, and neuropathic pain were male-specific predictors while taking antipsychotic medications; having COPD, malignancy, and liver disease were female-specific predictors. These sex-specific predictors should be fully investigated with more recent data when assessing the risk and benefit of COT in patients with HIV. Also, further studies should investigate the health outcomes associated with prescribing chronic opioids in patients with HIV.

## Supplementary Information

Supplementary Information.

## Data Availability

The data that support the findings of this study are available from Centers for Medicaid and Medicare Services but restrictions apply to the availability of these data, which were used under license for the current study, and so are not publicly available.
