# Supplementary material for: Predictors of chronic opioid therapy in Medicaid beneficiaries with HIV who initiated antiretroviral therapy
Source: Sci Rep. 2021 Jul 29;11:15503. doi: 10.1038/s41598-021-94690-8 (PMC8322087; doi:10.1038/s41598-021-94690-8)
Supplement: Supplementary file 1 — Supplementary Information. [file 41598_2021_94690_MOESM1_ESM.pdf]

Predictors of Chronic Opioid Therapy in Medicaid Beneficiaries with HIV who Initiated Antiretroviral Therapy

GYeon Oh[1,2,3], Emily S. Brouwer[4], Erin L. Abner[1,5,6], David W. Fardo[5,6], Patricia R. Freeman[2,3], Chris Delcher[2,3], and Daniela C. Moga[1,2,3,5*]

Affiliations

[1]Department of Epidemiology, University of Kentucky, Lexington, KY, USA,

[2]Department of Pharmacy Practice and Science, University of Kentucky, Lexington, KY, USA,

[3]Institute for Pharmaceutical Outcomes and Policy, University of Kentucky College of Pharmacy, Lexington, KY, USA,

[4]Takeda Pharmaceuticals, Cambridge, MA, USA

[5]Sanders-Brown Center on Aging, University of Kentucky, Lexington, KY, USA,

[6]Department of Biostatistics, University of Kentucky, Lexington, KY, USA

*Corresponding author

Daniela C. Moga

E-mail: daniela.moga@uky.edu

Telephone number: 859-323-9682

Mailing address: 241 Lee T.Todd Jr. Building, 789 S Limestone, Lexington KY 40536

Supplementary

Table 1S. Description of variables used in the study

Variables	ICD-9 codes
Cardiovascular disease[1)]	Myocardial infarction: 410.x, 412.x<br>Congestive heart failure: 398.91, 402.01, 402.11, 402.91, 404.01, 404.03, 404.11, 404.13, 404.91, 404.93, 425.4-425.9, 428.x<br>Peripheral vascular disease: 093.0, 437.3, 440.x, 441.x, 443.1-443.9, 47.1, 557.1, 557.9, V43.4<br>Cerebrovascular disease: 362.34, 430.x-438.x
Hypertension[1)]	401.x, 402.x-405.x
Dementia[1)]	290.x, 294.1, 331.2
COPD[1)]	416.8, 416.9, 490.x-505.x, 506.4, 508.1, 508.8
Any liver disease[1)]	070.22, 070.23, 070.32, 070.33, 070.44, 070.54, 070.6, 070.9, 570.x, 571.x, 573.3, 573.4, 573.8, 573.9, V42.7, 456.0-456.2, 572.2-572.8
Diabetes[1)]	250.0-250.3, 250.8, 250.9, 250.4-250.7
Renal disease[1)]	403.01, 403.11, 403.91, 404.02, 404.03, 404.12, 404.13, 404.92, 404.93, 582.x, 583.0-583.7, 585.x, 586.x, 588.0, V42.0, V45.1, V56.x
Any malignancy[1)]	140.x-172.x, 174.x-195.8, 200.x-208.x, 238.6, including lymphoma and leukemia, except malignant neoplasm of skin
Metastatic solid tumor[1)]	196.x-199.x
Alcohol abuse[1)]	265.2, 291.1-291.3, 291.5-291.9, 303.0, 303.9, 305.0, 357.5, 425.5, 535.3, 571.0- 571.3, 980.x, V11.3
Substance use disorder[1)]	292.x, 304.x, 305.2- 305.9, V65.42
Hepatitis C infection[2)]	070.41, 070.44, 070.51, 070.54, V02.62
Depression[1)]	296.2, 296.3, 296.5, 300.4, 309.x, 311
Psychotic disease[1)]	293.8, 295.x, 296.04, 296.14, 296.44, 296.54, 297.x, 298.x
Back pain[3)]	721.3x– 721.9x, 722.2x, 722.30, 722.70, 722.80, 722.90, 722.32, 722.72, 722.82, 722.92, 722.33, 722.73, 722.83, 722.93, 724.xx, 737.1, 737.3, 738.4, 738.5, 739.2, 739.3, 739.4, 756.10, 756.11, 756.12, 756. 13, 756.19, 805.4, 805.8, 839.2, 839.42, 846, 846.0, 847.1, 847.3, 847.2, 847.9
Neck pain[3)]	721.0X, 721.1X, 722.0X, 722.31, 722.71, 722.81, 722.91, 723.XX, 839.0, 839.1, 847.0
Unclassified pain	Pelvic pain: 595.1, 625.8, 625.9<br>Abdominal pain: 564.1<br>General pain: 338.0, 338.21, 338.29, 338.4, 729.1, 780.96
Arthritis/joint pain[3)]	>=710 and <720 or >=725 and <740
Neuropathy pain	Postherpetic neuropathy: 053.10-053.14, 053.19<br>Diabetic Neuropathy: 250.60-250.63, 357.2<br>Neuropathy: 337.00, 337.09, 337.1, 355.1, 356.2, 356.3, 356.4, 356.8, 356.9, 357.1, 357.4, 357.5, 357.6, 357.7, 357.81, 357.89, 357.9, 724.3<br>Neuralgia: 350.1, 729.2<br>Surgically induced pain: 338.22, 338.28<br>Limb pain: 337.20, 337.21, 337.22, 337.29, 353.6, 354.4, 355.0, 355.2, 355.3, 355.4, 355.6, 355.71, 355.79, 355.8, 355.9, 729.5
Migraine/headache[3)]	>=346 and <347, or 307.81
Any fracture	800.xx-829.xx

Opportunistic infection[4)]	Salmonella septicemia: 003.1<br>Cryptosporidiosis: 007.2<br>Other specified protozoal intestinal diseases: 007.8<br>Tuberculosis (all sites): 010.0-018<br>Disease due to other mycobacteria: 031.x<br>Actinomycotic infections: 039.x<br>Progressive multifocal leukoencephalopathy: 046.3<br>Cytomegalovirus: 078.5<br>Candidiasis (various sites): 112.0-112<br>Coccidiomycosis (various sites): 114.x<br>Histoplasmosis: 115.x<br>Crytococcosis: 117.5<br>Toxoplasmosis: 130.0-130<br>Pneumocystis Carinii pneumonia: 136.3<br>Cryptococcal meningitis: 321.0<br>Pneumonia in cytomegalic inclusion disease: 484.1
Surgery history[5)]	Total Knee Arthroplasty: 27447<br>Total Hip Arthroplasty: 27130<br>Laparoscopic Cholecystectomoy: 47562, 47563, 47564<br>Open Cholecystectomy: 47600, 47605, 47610<br>Laparoscopic Appendectomy: 44970, 44979<br>Open Appendectomy: 44950, 44960<br>Cesarean Section: 59510, 59514, 59515<br>FESS: 31237, 31240, 31254, 31255, 31256, 31267, 31276, 31287, 31288<br>Cataract Surgery: 66982, 66983, 66984<br>TURP: 52601, 52612, 52614<br>Simple Mastectomy: 19301, 19302, 19303, 19180
Tobacco history	305.1
Any opioid use	GPI starting with 65
Benzodiazepine	Therapeutic class generic product identifier including Temazepam, Alprazolam, bromazepam, chlordiazepoxide, clobazam, clonazepam, Clorazepate, Diazepam, Estazolam, Flunitrazepam, Flurazepam, Halazepam, Ketazolam, Loprazolam, Lorazepam, Lormetazepam, Medazepam, Nitrazepam, Nordazepam, Oxazepam, Prazepam, Quazepam, Temazepam, Triazolam, Midazolam using Medispan
Gabapentinoid	GPI starting with '9654424400' '7260003000' '7299600230' '6254003000' '6256003020' (Gabapentin) and GPI starting with '7260005700' (pregabalin)
Non-opioid pain medication	Analgesics-anti-inflammatory: GPI starting with 66<br>Analgesics-nonnarcotic: GPI starting with 64
Antidepressant	GPI starting with 58
Antipsychotic	GPI starting with 59

ICD: International Classification of Disease, 9th Clinical Modification; GPI: Generic Product Identifiers; NDC: National drug codes

1) Quan H, Sundararajan V, Halfon P, Fong A, Burnand B, Luthi JC, et al. Coding algorithms for defining comorbidities in ICD-9-CM and ICD-10 administrative data. Med Care. 2005;43(11):1130-9.
2) Kramer JR, Davila JA, Miller ED, Richardson P, Giordano TP, El-Serag HB. The validity of viral hepatitis and chronic liver disease diagnoses in Veterans Affairs administrative databases. Aliment Pharmacol Ther. 2008;27(3):274-82.

3) Sullivan MD, Edlund MJ, Fan MY, Devries A, Brennan Braden J, Martin BC. Trends in use of opioids for non-cancer pain conditions 2000-2005 in commercial and Medicaid insurance plans: the TROUP study. Pain. 2008;138(2):440-9.
4) Gebo KA, Fleishman JA, Moore RD. Hospitalizations for metabolic conditions, opportunistic infections, and injection drug use among HIV patients: trends between 1996 and 2000 in 12 states. J Acquir Immune Defic Syndr. 2005;40(5):609-16.
5) Sun EC, Darnall BD, Baker LC, Mackey S. Incidence of and Risk Factors for Chronic Opioid Use Among Opioid-Naive Patients in the Postoperative Period. JAMA Intern Med. 2016;176(9):1286-93.

Table 2S. Comparing predictors associated with chronic opioid use in Medicaid beneficiaries with HIV using different models

	Without clustering		State clustering		Full Model	Reduced Model using LASSO	Reduced Model using Elastic Net
	Full Model	Reduced Model	Full Model	Reduced Model			
Age	1.02 (1.01,1.03)	1.02 (1.02, 1.03)	1.02 (1.01 1.03)	1.02 (1.01, 1.03)	1.00 (1.00, 1.01)	1.02 (1.01, 1.03)	1.02 (1.01, 1.03)
Female	1.06 (0.92, 1.22)		1.06 (0.82, 1.37)		1.01 (0.99, 1.02)		1.08 (0.84, 1.38)
Race							
Black vs. white	0.74 (0.61, 0.90)	0.78 (0.66, 0.92)	0.76 (0.62, 0.93)	0.77 (0.65, 0.91)	0.98 (0.96, 0.99)	0.76 (0.63, 0.90)	0.76 (0.64, 0.90)
Other vs. white	0.68 (0.43, 1.08)	0.70 (0.44, 1.10)	0.67 (0.58, 0.79)	0.68 (0.59, 0.78)	0.96 (0.93, 1.00)	0.68 (0.60, 0.77)	0.68 (0.59, 0.79)
Unknown vs. white	0.82 (0.57, 1.20)	0.85 (0.59, 1.22)	0.82 (0.67, 1.01)	0.84 (0.67, 1.05)	0.98 (0.95, 1.01)	0.83 (0.67, 1.03)	0.84 (0.67, 1.04)
State							
KY vs. MD	0.83 (0.57, 1.19)						
NC vs. MD	0.83 (0.71, 0.98)						
WA vs. MD	0.91 (0.71, 1.17)						
Cardiovascular disease	0.78 (0.61, 0.98)	0.79 (0.63, 0.99)	0.78 (0.64, 0.96)	0.77 (0.68, 0.88)	0.97 (0.95, 1.00)		0.77 (0.67, 0.88)
Hypertension	1.12 (0.93, 1.34)		1.11 (0.95, 1.30)		1.01 (0.99, 1.03)		
Dementia	0.88 (0.54, 1.44)		0.89 (0.58, 1.36)		0.99 (0.94, 1.03)		
COPD	1.17 (0.97, 1.40)		1.16 (0.90, 1.50)	1.18 (0.92, 1.51)	1.02 (1.00, 1.03)	1.13 (0.87, 1.48)	1.15 (0.86, 1.55)
Any liver disease	0.94 (0.73, 1.21)		0.93 (0.74, 1.16)		0.99 (0.97, 1.02)		
Diabetes	0.91 (0.71, 1.18)		0.91 (0.78, 1.05)	0.92 (0.82, 1.02)	0.99 (0.97, 1.01)		
Renal disease	0.85 (0.63, 1.15)		0.85 (0.58, 1.25)		0.98 (0.95, 1.01)		
Any malignancy	1.25 (0.91, 1.73)		1.26 (1.18, 1.35)	1.28 (1.23, 1.33)	1.03 (0.99, 1.06)		1.29 (1.25, 1.33)
Metastatic solid tumor	1.11 (0.53, 2.30)		1.10 (0.67, 1.83)		1.03 (0.94, 1.11)		
Hepatitis C infection	1.60 (1.26, 2.02)	1.53 (1.28, 1.84)	1.62 (1.41, 1.87)	1.55 (1.47, 1.64)	1.06 (1.09, 1.04)	1.49 (1.41, 1.57)	1.55 (1.47, 1.64)
Depression	0.99 (0.82, 1.18)		0.98 (0.75, 1.30)		1.00 (0.98, 1.02)		
Psychotic disorder	0.71 (0.53, 0.96)	0.72 (0.55, 0.95)	0.71 (0.63, 0.80)	0.70 (0.62, 0.79)	0.97 (0.94, 0.99)		0.73 (0.65, 0.81)
Migraine/headache	0.88 (0.49, 1.57)		0.86 (0.61, 1.21)		0.98 (0.93, 1.03)		
Any fracture	0.70 (0.45, 1.10)		0.70 (0.57, 0.87)	0.70 (0.55, 0.89)	0.96 (0.92, 1.01)		0.70 (0.55, 0.89)
Surgery history	1.04 (0.56, 1.93)		1.04 (0.77, 1.39)		1.01 (0.95, 1.07)		
Opportunistic infection	1.06 (0.89, 1.26)		1.05 (0.98, 1.13)	1.04 (0.98, 1.11)	1.00 (0.99, 1.02)		
Alcohol abuse	0.85 (0.67, 1.09)		0.85 (0.69, 1.03)	0.84 (0.66, 1.05)	0.98 (0.96, 1.00)		0.83 (0.65, 1.05)
Substance use disorder	1.58 (1.33, 1.87)	1.59 (1.35, 1.86)	1.62 (1.22, 2.16)	1.62 (1.25, 2.12)	1.05 (1.03, 1.07)	1.50 (1.19, 1.90)	1.61 (1.26, 2.04)
Tobacco use disorder	0.87 (0.69, 1.10)		0.86 (0.74, 1.00)	0.86 (0.73, 1.02)	0.98 (0.96, 1.01)		

Back pain	1.45 (1.17, 1.79)	1.46 (1.19, 1.80)	1.44 (1.11, 1.88)	1.46 (1.10, 1.93)	1.05 (1.03, 1.07)	1.43 (1.07, 1.93)	1.45 (1.09, 1.92)
Neck pain	1.02 (0.72, 1.45)		1.02 (0.88, 1.17)		1.00 (0.97, 1.04)		
Arthritis	1.12 (0.93, 1.35)		1.11 (0.98, 1.26)	1.13 (1.01, 1.25)	1.01 (0.99, 1.03)	1.07 (0.97, 1.19)	1.12 (1.01, 1.24)
Neuropathy pain	1.19 (0.95, 1.49)	1.24 (1.01, 1.51)	1.18 (1.04, 1.33)	1.17 (1.05, 1.30)	1.02 (1.00, 1.04)	1.15 (1.03, 1.29)	1.16 (1.04, 1.30)
Unclassified pain	1.05 (0.77, 1.43)		1.04 (0.91, 1.20)		1.01 (0.98, 1.04)		
Number of medication (vs. none)							
1-4medications	1.35 (1.16, 1.57)	1.36 (1.17, 1.58)	1.35 (1.29, 1.41)	1.34 (1.29, 1.40)	1.02 (1.01, 1.04)	1.34 (1.29, 1.40)	1.35 (1.30, 1.41)
5+ medications	1.71 (1.37, 2.14)	1.74 (1.40, 2.16)	1.73 (1.44, 2.08)	1.71 (1.40, 2.09)	1.06 (1.03, 1.08)	1.66 (1.36, 2.03)	1.72 (1.42, 2.09)
Non-opioid pain medication	1.14 (0.98, 1.33)	1.18 (1.01, 1.37)	1.15 (0.94, 1.41)	1.16 (0.96, 1.40)	1.01 (1.00, 1.03)	1.16 (0.95, 1.42)	1.16 (0.95, 1.42)
Benzodiazepine	1.64 (1.35, 1.99)	1.62 (1.34, 1.96)	1.62 (1.49, 1.76)	1.62 (1.48, 1.77)	1.06 (1.04, 1.09)	1.60 (1.46, 1.76)	1.62 (1.49, 1.76)
Gabapentinoid	1.33 (1.05, 1.69)	1.33 (1.05, 1.68)	1.33 (1.21, 1.46)	1.33 (1.21, 1.45)	1.04 (1.01, 1.07)	1.33 (1.18, 1.49)	1.33 (1.19, 1.49)
Antidepressant	1.31 (1.11, 1.54)	1.35 (1.16, 1.56)	1.31 (1.06, 1.62)	1.31 (1.10, 1.56)	1.03 (1.01, 1.04)	1.32 (1.09, 1.60)	1.33 (1.11, 1.58)
Antipsychotic	1.08 (0.88, 1.33)		1.09 (0.96, 1.24)	1.09 (0.99, 1.21)	1.01 (0.99, 1.03)		
Any opioid use	1.87 (1.61, 2.17)	1.87 (1.62, 2.15)	1.85 (1.59, 2.15)	1.86 (1.61, 2.13)	1.06 (1.05, 1.08)	1.84 (1.61, 2.10)	1.84 (1.60, 2.11)

Table 3S. Comparing predictors associated with chronic opioid use in male Medicaid beneficiaries with HIV using different models

	Without clustering		State clustering		Full Model	Reduced Model using LASSO	Reduced Model using Elastic Net
	Full Model	Reduced Model	Full Model	Reduced Model			
Age	1.02 (1.01,1.03)	1.02 (1.01, 1.03)	1.02 (1.00 1.03)	1.02 (1.00, 1.03)	1.00 (1.00, 1.00)	1.02 (1.00, 1.03)	1.02 (1.01, 1.03)
Race							
Black vs. white	0.89 (0.68, 1.17)		0.76 (0.65, 0.90)	0.76 (0.68, 0.85)	0.98 (0.96, 1.00)		
Other vs. white	0.97 (0.55, 1.73)		0.93 (0.81, 1.06)	0.93 (0.81, 1.07)	0.99 (0.94, 1.04)		
Unknown vs. white	0.83 (0.51, 1.36)		0.74 (0.53, 1.03)	0.74 (0.53, 1.04)	0.97 (0.95, 1.00)		
State							
KY vs. MD	1.25 (0.79, 1.99)	1.31 (0.85, 2.04)					
NC vs. MD	0.95 (0.76, 1.19)	0.94 (0.76, 1.17)					
WA vs. MD	1.26 (0.92, 1.73)	1.34 (1.03, 1.76)					
Cardiovascular disease	0.77 (0.56, 1.06)		0.77 (0.64, 0.93)	0.78 (0.64, 0.94)	0.97 (0.95, 1.00))		
Hypertension	1.19(0.92, 1.54)		1.16 (0.91, 1.48)	1.16 (0.90, 1.48)	1.01 (0.99, 1.04)		
Dementia	0.90(0.48, 1.69)		0.92 (0.75, 1.14)	0.92 (0.72, 1.18)	0.99 (0.93, 1.05)		
COPD	1.07 (0.81, 1.43)		1.06 (0.75, 1.51)		1.01 (0.98, 1.04)		
Any liver disease	1.09 (0.78, 1.53)		1.07 (0.69, 1.67)		1.01 (0.98, 1.04)		
Diabetes	0.71 (0.49, 1.03)		0.69 (0.50, 0.94)	0.69 (0.50, 0.96)	0.97 (0.93, 1.00)		
Renal disease	0.90 (0.61, 1.32)		0.89 (0.68, 1.16)	0.90 (0.70, 1.14)	0.99 (0.95, 1.02)		
Any malignancy	1.00 (0.64, 1.58)		1.00 (0.95, 1.05)		1.00 (0.96, 1.04)		
Metastatic solid tumor	2.39 (0.93, 6.19)		2.37 (1.28, 4.39)	2.41 (1.29, 4.50)	1.14 (1.01, 1.27)		
Hepatitis C infection	1.48 (1.08, 2.03)	1.51(1.17, 1.94)	1.48 (1.21, 1.81)	1.54 (1.44, 1.64)	1.04 (1.01, 1.08)	1.44 (1.34, 1.55)	1.50 (1.41, 1.59)
Depression	1.01(0.77, 1.31)		0.98 (0.62, 1.55)		1.00 (0.97, 1.02)		
Psychotic disorder	0.64(0.42, 0.99)	0.62 (0.41, 0.92)	0.64 (0.51, 0.81)	0.63 (0.56, 0.71)	0.96 (0.93, 1.00)		
Migraine/headache	0.90 (0.37, 2.19)		0.85 (0.42, 1.75)		0.98 (0.90, 1.07)		
Any fracture	0.72 (0.41, 1.24)		0.71 (0.43, 1.15)	0.70 (0.43, 1.17)	0.97 (0.92, 1.02)		
Surgery history	1.46 (0.61, 3.46)		1.42 (0.96, 2.12)	1.44 (1.04, 1.99)	0.95 (1.04, 1.14)		
Opportunistic infection	1.03 (0.81, 1.31)		1.00 (0.86, 1.18)		1.00 (0.98, 1.02)		
Alcohol abuse	0.98 (0.72, 1.34)		0.96 (0.81, 1.14)		1.00 (0.97, 1.03)		
Substance use disorder	1.57 (1.23, 2.00)	1.54 (1.22, 1.93)	1.61 (1.34, 1.93)	1.59 (1.38, 1.84)	1.05 (1.02, 1.07)	1.43 (1.15, 1.77)	1.49 (1.18,1.87)
Tobacco use disorder	0.85 (0.62, 1.18)		0.84 (0.74, 0.97)	0.85 (0.74, 0.98)	0.98 (0.95, 1.01)		
Back pain	1.69 (1.27, 2.24)	1.80 (1.37, 2.37)	1.65 (1.06, 2.58)	1.65 (1.02, 2.66)	1.07 (1.03, 1.10)	1.73 (1.06, 2.83)	1.52 (1.10, 2.09)

Neck pain	1.29 (0.82, 2.04)		1.28 (0.95, 1.74)	1.29 (0.95, 1.74)	1.04 (0.99, 1.09)		
Arthritis	1.07 (0.83, 1.39)		1.04 (0.97, 1.13)	1.05 (0.97, 1.13)	1.01 (0.98, 1.03)	1.06 (0.96, 1.17)	
Neuropathy pain	1.61 (1.19, 2.18)	1.69 (1.30, 2.19)	1.61 (1.37, 1.90)	1.62 (1.38, 1.90)	1.06 (1.02, 1.09)	1.57 (1.34, 1.84)	1.25 (1.16, 1.34)
Unclassified pain	1.43 (0.87, 2.35)		1.45 (1.09, 1.94)	1.46 (1.08, 1.98)	1.05 (0.99, 1.11)		
Number of medication (vs. none)							
1-4medications	1.39 (1.13, 1.72)	1.40 (1.13, 1.73)	1.39 (1.26, 1.53)	1.39 (1.27, 1.52)	1.03 (1.01, 1.04)	1.38 (1.25, 1.53)	1.34 (1.28, 1.41)
5+ medications	1.71 (1.27, 2.32)	1.66 (1.24, 2.23)	1.75 (1.50, 2.05)	1.76 (1.52, 2.02)	1.05 (1.02, 1.08)	1.64 (1.40, 1.92)	1.69 (1.39, 2.06)
Non-opioid pain medication	1.09 (0.87, 1.36)		1.12 (0.86, 1.47)	1.12 (0.85, 1.47)	1.01 (0.99, 1.03)		
Benzodiazepine	1.68 (1.29, 2.19)	1.70 (1.31, 2.20)	1.67 (1.45, 1.93)	1.67 (1.45, 1.93)	1.06 (1.03, 1.10)	1.70 (1.48, 1.95)	1.71 (1.56, 1.87)
Gabapentinoid	1.23 (0.89, 1.70)		1.22 (1.02, 1.47)	1.22 (1.03, 1.44)	1.02 (0.99, 1.06)		
Antidepressant	1.27 (1.01, 1.59)	1.32 (1.08, 1.62)	1.30 (1.02, 1.65)	1.28 (1.06, 1.54)	1.03 (1.00, 1.05)	1.32 (1.16, 1.49)	1.41 (1.19, 1.67)
Antipsychotic	0.95 (0.70, 1.28)		0.97 (0.68, 1.38)		1.00 (0.97, 1.02)		
Any opioid use	1.76 (1.44, 2.16)	1.82 (1.49, 2.20)	1.79 (1.43, 2.25)	1.80 (1.42, 2.27)	1.06 (1.04, 1.08)	1.81 (1.51, 2.18)	1.93 (1.69, 2.20)

Table 4S. Comparing predictors associated with chronic opioid use in female Medicaid beneficiaries with HIV using different models

	Without clustering		State clustering		Full Model	Reduced Model using LASSO	Reduced Model using Elastic Net
	Full Model	Reduced Model	Full Model	Reduced Model			
Age	1.03 (1.02, 1.04)	1.03 (1.02, 1.04)	1.03(1.02, 1.03)	1.03 (1.02, 1.03)	1.00 (1.00, 1.00)	1.03 (1.02, 1.04)	1.03 (1.02, 1.03)
Race							
Black vs. white	0.62 (0.47, 0.82)	0.61 (0.46, 0.81)	0.68 (0.50, 0.93)	0.67 (0.49, 0.91)	0.97 (0.94, 0.99)	0.66 (0.50, 0.87)	
Other vs. white	0.47 (0.22, 1.01)	0.47 (0.22, 1.02)	0.46 (0.39, 0.54)	0.46 (0.39, 0.55)	0.93 (0.88, 0.99)	0.45 (0.40, 0.50)	
Unknown vs. white	0.87 (0.49, 1.55)	0.86 (0.49, 1.53)	0.90 (0.74, 1.11)	0.90 (0.73, 1.11)	0.99 (0.93, 1.04)	0.89 (0.75, 1.06)	
State							
KY vs. MD	0.48 (0.25, 0.90)	0.49 (0.26, 0.92)					
NC vs. MD	0.74 (0.59, 0.92)	0.71 (0.57, 0.89)					
WA vs. MD	0.56 (0.34, 0.91)	0.58 (0.36, 0.94)					
Cardiovascular disease	0.77 (0.54, 1.11)		0.79 (0.64, 0.97)	0.79 (0.62, 1.00)	0.98 (0.95, 1.01)		
Hypertension	1.06 (0.82, 1.38)		1.06 (0.97, 1.15)	1.08 (1.04, 1.12)	1.01 (0.98, 1.03)		
Dementia	0.84 (0.36, 1.95)		0.84 (0.33, 2.10)		0.97 (0.90, 1.06)		
COPD	1.25 (0.97, 1.60)		1.26 (0.97, 1.63)	1.25 (0.94, 1.66)	1.02 (1.00, 1.05)	1.21 (0.88, 1.67)	
Any liver disease	0.78 (0.53, 1.15)		0.77 (0.63, 0.94)	0.75 (0.62, 0.90)	0.97 (0.93, 1.01)		
Diabetes	1.19 (0.84, 1.69)		1.17 (0.77, 1.80)		1.02 (0.98, 1.05)		
Renal disease	0.77 (0.47, 1.25)		0.76 (0.45, 1.30)	0.77 (0.46, 1.26)	0.97 (0.92, 1.01)		
Any malignancy	1.63 (1.01, 2.62)		1.62 (1.24, 2.11)	1.61 (1.25, 2.07)	1.06 (1.01, 1.12)		
Metastatic solid tumor	0.46 (0.14, 1.59)		0.49 (0.29, 0.82)	0.48 (0.30, 0.76)	0.93 (0.82, 1.04)		
Hepatitis C infection	1.78 (1.25, 2.54)	1.53 (1.16, 2.02)	1.86 (1.61, 2.15)	1.86 (1.60, 2.16)	1.08 (1.04, 1.13)	1.52 (1.30, 1.78)	1.57 (1.32, 1.86)
Depression	0.97 (0.75, 1.26)		0.98 (0.83, 1.16)		1.00 (0.97, 1.02)		
Psychotic disorder	0.75 (0.50, 1.13)		0.76 (0.60, 0.96)	0.74 (0.60, 0.92)	0.97 (0.93, 1.01)		
Migraine/headache	0.93 (0.43, 2.01)		0.91 (0.72, 1.16)		0.98 (0.92, 1.05)		
Any fracture	0.67 (0.31, 1.48)		0.66 (0.39, 1.13)	0.62 (0.39, 0.99)	0.95 (0.89, 1.02)		
Surgery history	0.85 (0.34, 2.10)		0.83 (0.62, 1.12)	0.82 (0.60, 1.12)	0.98 (0.91, 1.06)		
Opportunistic infection	1.08 (0.85, 1.39)		1.08 (0.88, 1.32)		1.01 (0.98, 1.03)		
Alcohol abuse	0.70 (0.47, 1.03)	0.67 (0.46, 0.98)	0.68 (0.32, 1.48)		0.96 (0.99, 0.92)		
Substance use disorder	1.61 (1.26, 2.04)	1.57 (1.24, 1.98)	1.67 (1.08, 2.60)	1.58 (1.12, 2.22)	1.06 (1.03, 1.08)	1.50 (1.10, 2.03)	1.49 (1.09, 2.04)
Tobacco use disorder	0.85 (0.59, 1.22)		0.83 (0.65, 1.06)	0.81 (0.57, 1.15)	0.98 (0.95, 1.01)		
Back pain	1.22 (0.88, 1.69)		1.22 (1.07, 1.39)	1.21 (1.06, 1.38)	1.03 (0.99, 1.06)		

Neck pain	0.70 (0.39, 1.24)		0.70 (0.36, 1.36)	0.70 (0.36, 1.37)	0.96 (0.91, 1.01)		
Arthritis	1.20 (0.91, 1.58)		1.18 (0.95, 1.47)	1.09 (0.91, 1.32)	1.02 (0.99, 1.05)		
Neuropathy pain	0.80 (0.56, 1.14)		0.80 (0.57, 1.12)		0.98 (0.94, 1.01)		
Unclassified pain	0.89 (0.59, 1.32)		0.89 (0.72, 1.10)	0.87 (0.70, 1.10)	0.99 (0.95, 1.03)		
Number of medication (vs. none)							
1-4medications	1.30 (1.04, 1.63)	1.30 (1.04, 1.62)	1.32 (1.30, 1.33)	1.33 (1.32, 1.33)	1.02 (1.00, 1.04)	1.31 (1.30, 1.32)	1.29 (1.28, 1.30)
5+ medications	1.74 (1.24, 2.43)	1.75 (1.27, 2.43)	1.78 (1.25, 2.54)	1.82 (1.29, 2.58)	1.07 (1.03, 1.10)	1.77 (1.24, 2.51)	1.73 (1.22, 2.45)
Non-opioid pain medication	1.20 (0.96, 1.49)		1.20 (1.01, 1.42)	1.20 (1.00, 1.45)	1.02 (1.00, 1.04)	1.23 (1.01, 1.48)	1.21 (0.99, 1.46)
Benzodiazepine	1.66 (1.24, 2.22)	1.61 (1.21, 2.14)	1.64 (1.59, 1.69)	1.63 (1.60, 1.66)	1.07 (1.03, 1.10)	1.59 (1.58, 1.59)	1.70 (1.61, 1.78)
Gabapentinoid	1.47 (1.03, 2.10)	1.49 (1.06, 2.10)	1.47 (0.98, 2.20)	1.41 (0.97, 2.05)	1.06 (1.02, 1.11)	1.44 (1.01, 2.06)	1.44 (0.99, 2.09)
Antidepressant	1.35 (1.07, 1.72)	1.41 (1.14, 1.75)	1.34 (1.06, 1.70)	1.33 (1.10, 1.60)	1.03 (1.01, 1.05)	1.37 (1.15, 1.63)	1.42 (1.20, 1.67)
Antipsychotic	1.19 (0.89, 1.60)		1.20 (1.06, 1.36)	1.19 (1.06, 1.35)	1.02 (0.99, 1.05)		
Any opioid use	2.02 (1.63, 2.50)	2.09 (1.70, 2.56)	2.00 (1.83, 2.19)	2.01 (1.82, 2.21)	1.07 (1.05, 1.09)	1.96 (1.73, 2.21	1.97 (1.75, 2.22)
